# Synthesis, structure, and opto-electronic properties of organic-based nanoscale heterojunctions

**DOI:** 10.1186/1556-276X-6-238

**Published:** 2011-03-18

**Authors:** Bohuslav Rezek, Jan Čermák, Alexander Kromka, Martin Ledinský, Pavel Hubík, Jiří J Mareš, Adam Purkrt, Vĕra Cimrová, Antonín Fejfar, Jan Kočka

**Affiliations:** 1 Institute of Physics ASCR, v.v.i., Cukrovarnická 10, 16200 Prague 6, Czech Republic; 2 Institute of Macromolecular Chemistry ASCR, v.v.i., Heyrovského nám. 2, 16206 Prague 6, Czech Republic

## Abstract

Enormous research effort has been put into optimizing organic-based opto-electronic systems for efficient generation of free charge carriers. This optimization is mainly due to typically high dissociation energy (0.1-1 eV) and short diffusion length (10 nm) of excitons in organic materials. Inherently, interplay of microscopic structural, chemical, and opto-electronic properties plays crucial role. We show that employing and combining advanced scanning probe techniques can provide us significant insight into the correlation of these properties. By adjusting parameters of contact- and tapping-mode atomic force microscopy (AFM), we perform morphologic and mechanical characterizations (nanoshaving) of organic layers, measure their electrical conductivity by current-sensing AFM, and deduce work functions and surface photovoltage (SPV) effects by Kelvin force microscopy using high spatial resolution. These data are further correlated with local material composition detected using micro-Raman spectroscopy and with other electronic transport data. We demonstrate benefits of this multi-dimensional characterizations on (i) bulk heterojunction of fully organic composite films, indicating differences in blend quality and component segregation leading to local shunts of photovoltaic cell, and (ii) thin-film heterojunction of polypyrrole (PPy) electropolymerized on hydrogen-terminated diamond, indicating covalent bonding and transfer of charge carriers from PPy to diamond.

## Introduction

Electronic devices nowadays are commonly based on inorganic semiconductors (e.g., silicon or germanium) and, thus, face the problem of high-cost industrial processes (high-vacuum technology, clean rooms, or high-purity source materials). A possible way to reduce costs is the use of organic materials with semiconducting or metallic properties. Although the first report on the conductivity of doped polypyrrole (PPy) was published in 1963 [1], the breakthrough is attributed to the studies on doped polyacetylene since 1977 by Heeger, Shirakawa, and MacDiarmid [2], for which they were awarded the Nobel prize in 2000. Since then organic semiconductors have followed similar scientific evolution as inorganic ones (all-polymer field effect transistor in 1994 [3], organic integrated circuit by the Philips company (1998)) until recently, which have seen application of organic displays in cell phones, and the start of commercial production of organic photovoltaic (PV) cells.

**Figure 1:**
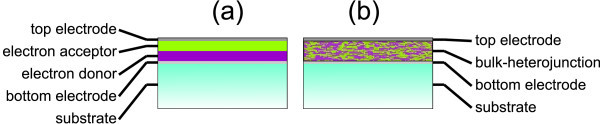
**Schematic cross-sectional drawings of two main organic PV cell designs**: **(a) **double-layer junction and **(b) **bulk-heterojunction.

PV effect in organic materials is different from inorganic ones. The binding energy between photo-excited electron-hole pair is strong due to the low dielectric constant, typically in the range of 0.1-1 eV. Therefore, the excitons are not dissociated by thermal energy, which is approximately 26 meV at room temperature. Additional driving force is needed, which can be supplied by introducing a layer of a second organic material (the so-called double-layer cell, Figure 1a). Typical PV power conversion efficiencies of such devices are not higher than 0.1% [4,5] because of the short exciton diffusion length (around 10 nm [6]) compared to the total film thickness needed for efficient light harvesting (100-200 nm).

Significant improvement emerged with the bulk-heterojunction design [7]. In the bulk-heterojunction design both materials (donor and acceptor) are prepared as a microscopic interpenetrating network (Figure 1b). Therefore, wherever the excitons are generated, an interface with the other material is always closer than the exciton diffusion length (in an ideal case). The remaining task is to ensure efficient charge carrier transport to electrodes. So far the best reported PV efficiency is the attainment of reaching 7.4% [8].

For a long time, electronics has been the domain of inorganic materials. Even though electronic behavior of organic materials has been known for several decades, their applications are still limited. So far, the research and development activity of organic and inorganic electronics has been strictly divided, although their combination might be fruitful. An example of promising organic-inorganic (hybrid) systems is the dye-sensitized PV cell. In this so-called Grätzel cell [9], a photoexcited organic dye gives electrons to the electrode via porous inorganic TiO_2_. The missing electron is returned from the surrounding electrolyte, which restores the original state at the counter electrode. Although the PV power conversion of this type of PV cells is relatively high (10-12%), their wider application is limited by the need of an electrolyte, which is commonly in liquid form.

Another example of an organic-inorganic system, which is extensively studied, is diamond in combination with organics, e.g., fullerene [10,11]. Such systems are highly promising for bio-sensoric or opto-electronic applications, as a charge transport between the two materials is observed. However, the basic electronic properties at their interface still need to be fully understood.

From the electronic point of view, diamond is a wide bandgap (5.5 eV) semiconductor, which in its intrinsic form is electrically insulating. Apart from the bulk conductivity induced by doping [12], intrinsic diamond exhibits a special phenomenon of surface conductivity when a thin (10-20 nm) electrically conductive (*p*-type) layer is formed at the surface [13-15]. It is observed under ambient conditions when the diamond surface is terminated by hydrogen atoms. Various electrically conductive areas or channels can be patterned on the surface by changing the surface termination [16,17]. Hydrogen termination can also be used as a starting surface for grafting of more complex organic molecules on diamond [10,18-20].

In this study, we have chosen PPy because of its wide universality as a model of a chemically and optically sensitive organic dye. Its polymerization can be achieved by electrooxidation from a solvent [21], chemical vapor deposition [22], UV irradiation [23], or chemical polymerization [24,25]. PPy is a well known yet still remains under intensive study in many fields of applications, like sensors [26-28], biosensors [29,30], fuel cells [31,32], corrosion protection [33], or rechargeable batteries [34,35].

Organic-based electronic as well as optoelectronic devices are commonly made from several compounds and their mutual electronic cooperation at microscale is of key importance for the device properties. Therefore, revealing and understanding of microscopic structural, chemical, electronic, and optoelectronic properties is crucial for their further improvements. Such challenging task may be fulfilled by techniques based on local probe scanning. One of the prominent methods employing scanning probe is atomic force microscopy (AFM) with its diverse regimes of operation [36]. In AFM, a sharp tip mounted on a flexible lever (the so-called can-tilever) can detect both morphologic and electronic information with high lateral resolution.

When the tip is in contact with the studied surface, a bias voltage can be applied between the sample and the AFM tip, and the induced electric current is dependent on the local conductivity at the position of the tip. As a result, maps of both topography and local conductivity are obtained at once in this so-called current-sensing AFM regime (CS-AFM) [37]. Applying this mode on organic materials is rather difficult, however, as the soft organic materials can be easily modified by the sharp AFM tip (radius typically 10-30 nm) under the applied force of typically several nN or more.

Application of an AC voltage between the sample and the AFM tip when the tip is at a certain distance from the surface creates a modulated electrostatic force that makes the cantilever oscillate because of contact potential difference. These oscillations can be minimized to zero by applying an additional DC voltage to compensate the potential difference. If the tip surface potential is calibrated, then the absolute values of the surface potential (and then the work function) can be obtained. This is the principle of the so-called Kelvin force microscopy (KFM) [16].

In spite of being a powerful tool for resolving microscopic structural, mechanical, and electronic properties, AFM had not been able to provide direct chemical contrast until very recently [38]. Such chemical detection in AFM makes use of adhesion differences on atomic scale and was, thus so far, applicable only to atomically flat surfaces. Spatially resolved chemical information can be obtained in more straightforward way by micro-Raman spectroscopy mapping, where the focused laser beam plays the role of the scanning probe. Although it is an optical method, the resolution can be satisfactory at sub-micrometer scale [39]. The resolution can be further enhanced using special metal tip in the so-called tip-enhanced Raman spectroscopy [40]. This method works well on resonant systems such as in the case of carbon nanotubes [41], but its general application is still highly challenging.

In this study, we employ and combine advanced scanning probe techniques as well as macroscopic characteristics to provide us significant insights into the correlation of microscopic structural, chemical, and opto-electronic properties of organic-based heterojunctions. We demonstrate benefits of such multi-dimensional characterizations on (i) bulk heterojunctions of fully organic composite films using fullerenes as electron acceptors, indicating differences in blend quality and component segregation leading to local shunts of PV cell [42,43], and (ii) thin-film heterojunction of PPy electro-polymerized on hydrogen-terminated diamond, indicating covalent bonding and enhanced exciton dissociation in such systems [44-47].

## Basic organic bulk heterojunction

Basic type of organic bulk heterojunction was based on fullerene C_60 _as electron acceptor. Composite blends made of poly[(2,7-(9,9-dihexa)fluorence)-co-(1,4-(2,5-didecylaminoketo) phenylene)] (VYP-120, developed at the Institute of Macro-molecular Chemistry, ASCR, v.v.i.) and fullerene C_60 _(Sigma Aldrich) were spincoated on indium tin oxide (ITO)-covered glass substrates and dried at 50°C under vacuum for 4 h. For characterizing the PV performance of the thin film, top Al electrodes were evaporated through a shadow mask.

**Figure 2:**
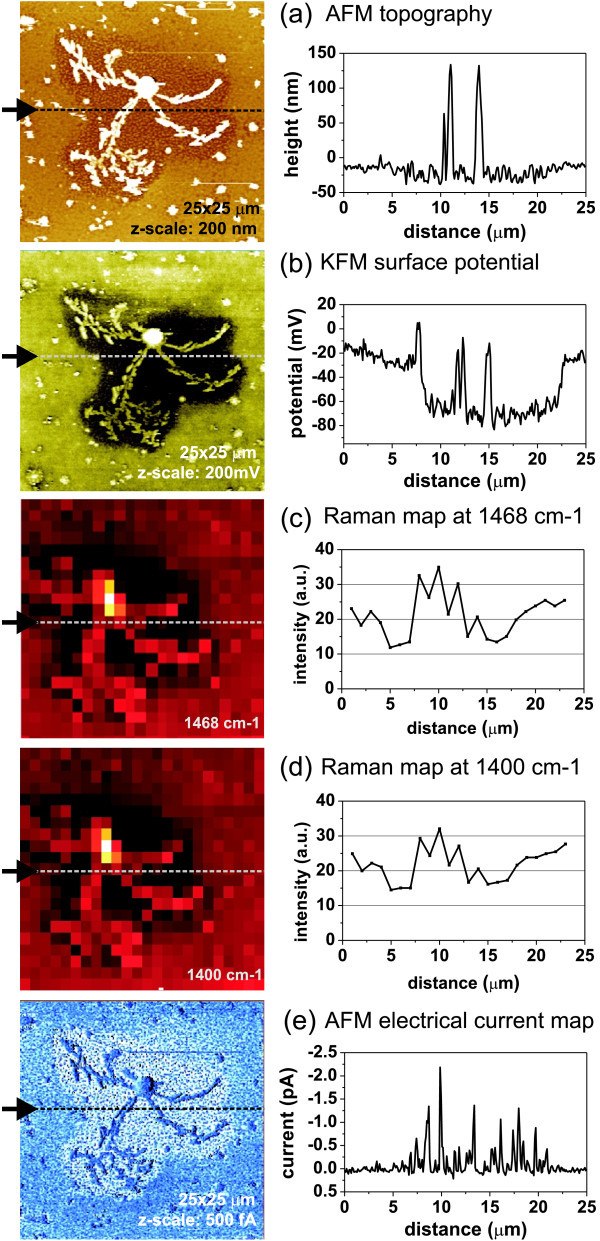
**Multidimensional microscopic characteristics (images and spatial profiles) of the organic heterostructure blend made of fullerene C_60 _and poly[(2,7-(9,9-dihexa)fluorene)-co-(1,4-(2,5-didecylaminoketo) phenylene)]**: **(a) **tapping-mode AFM surface morphology, **(b) **KFM surface potential, **(c) **micro-Raman intensity at 1468 cm^-1^, **(d) **micro-Raman intensity at 1400 cm^-1^, and **(e) **local conductivity as measured by CS-AFM (negative bias voltage applied on the sample). Positions of the profiles in the images are indicated by the dashed lines and arrows.

In spite of observed quenching of photoluminescence compared to polymer layer without the C_60_, the composite layer exhibited low PV power conversion efficiency (*I*_sc _~ 2 nA, *V*oc ~ 5 mV, *η *~ 0.06%) [42]. AFM morphology (Figure 2a) revealed a relatively flat and smooth surface (RMS roughness: 4 nm) which was covered with two types of clusters (lateral size either 100 nm or several *μ*m) and dendrites (more than 10 *μ*m). Electrical potential map obtained by KFM at the same area is shown in Figure 2b. KFM detected the highest surface potential (up to 50 mV) in the central part of the dendrite and the lowest surface potential (as low as -150 mV) in its immediate surrounding. Farther surroundings exhibit the potential between these two values. Typical value is around *- *30 mV. Also fluctuations related with small clusters are seen in topography. As the layer is made of two materials, the observed variations in surface potential most likely correspond to variations in local chemical composition. The two extreme potential levels may correspond to individual materials while the intermediate potential is related to the blend.

This assertion is proven by micro-Raman spectroscopy and mapping. The spectrum collected on the dendrite showed a sharp peak at 1468 cm^-1^, which was attributed to fullerene C_60_, as well as a broad band at 1400 cm^-1 ^[42,48]. Similar spectra, although with lower peak intensity, were detected on the small clusters in the surroundings. The map and the spatial profile of C_60 _characteristic Raman scattering peak at 1468 cm^-1 ^are shown in Figure 2c. The image reveals higher concentration of C_60 _at the dendrite and low C_60 _Raman signal in its close surroundings.

Spectrum detected in the dendrite vicinity did not show the sharp peak, only a broad band around 1400 cm^-1 ^[42]. Similar spectra were obtained on the smooth surface in the farther surroundings. This band is not present in typical C_60 _Raman spectrum [48]. Hence, we attribute it to a photoluminescence (PL) background due to Raman laser excitation (note that it cannot be directly compared with usual PL spectra). Spatially resolved map and the profile of Raman intensity at the 1400 cm^-1 ^band are shown in Figure 2d. It is noteworthy that both Raman maps are qualitatively quite similar. Yet, quantitative comparison of the spatial profiles shows differences: the C_60 _signal is higher on the dendrite and small clusters, while the PL intensity prevails over C_60 _on the smooth surface and the very vicinity of the dendrite. We suggest that the map of sharp peak at 1458 cm^-1 ^is related with segregated crystallized C_60 _on the dendrite and on small clusters in the surroundings. The broad band at around 1400 cm^-1 ^may be attributed to C_60 _that is highly dissolved in the blend. This assertion is supported also by the AFM and KFM data.

In both Raman maps, there is a minimum in the vicinity of the dendrite. This is not effect of thickness as the AFM shows even surface compared to surroundings. On the other hand, there is clear difference in the work function detected by KFM. This indicates that this area consists predominantly of the conductive polymer. In such a case, the dendrite vicinity is not of heterostructural nature, and the conductive polymer may electrically shunt the device.

To resolve the above effect, we characterized local electronic transport properties of the organic film by CS-AFM, so that its integrity was preserved, and its surface morphology was the same as in tapping mode. The image of local conductivity (Figure 2e: note that the applied bias voltage is negative) shows that the dendrite and farther surroundings are electrically insulating. Conductivity in the close vicinity of the dendrite (area lacking C_60_) is an order of magnitude higher. This observation corroborates the conclusion that such features can cause electrical shunting of the organic film and reduce its power conversion efficiency.

### Advanced organic bulk heterojunction

Based on the result with pure fullerene, more advanced bulk heterojunctions were prepared, with fullerene derivatives being used as electron acceptors: [6,6]-thienylC61 butyric acid methyl ester ([60]ThCBM), and [6,6]-thienylC71 butyric acid methyl ester([70]ThCBM). Poly(3-hexylthiophene-2,5-diyl) (P3HT) was used as electron donor. The blends were prepared in an inert atmosphere on ITO-covered glass substrates with a hole-conducting polymer (PEDOT:PSS) by spin coating from a 2.5% chlorobenzene solution [43]. Both blends were annealed at 125°C (the annealing improved the PV efficiency more than twice compared with non-annealed blends). Resulting thickness of the films was 140 nm, as measured by KLA-Tencor P-10 profiler. LiF (2 nm) and Al (60 nm) were thermally evaporated to make the second contact. Active area of the prepared PV cells was 6.5 × 2.5 mm^2^.

**Figure 3:**
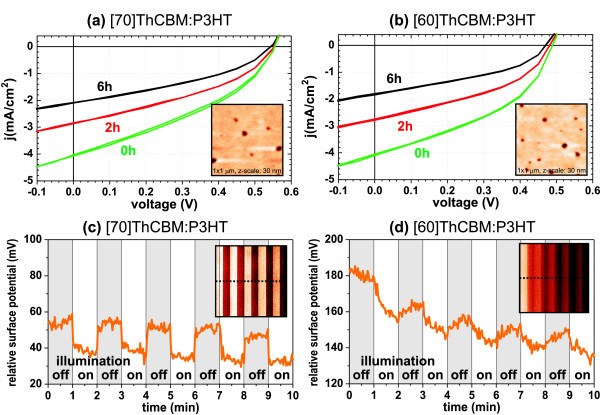
**Macroscopic and microscopic opto-electronic characteristics of the organic blends**. **(a, b) **Macroscopically measured current-voltage characteristics of the blend films under illumination (white light, 80 mW) in the nitrogen atmosphere (0 h) and after exposure to ambient air for 2 and 6 h. The inset images in **(a, b) **show AFM topography images of the blend films (1 × 1 *μ*m^2^, height scale 30 nm). **(c, d) **Cross sections of the KFM images (shown as insets, 1 × 1 *μ*m^2^, potential scale 40 mV) under repeated on/off switching of illumination.

Figure 3a, b shows macroscopic (with both top and bottom planar contacts) *I*(*V*) characteristics of the two blend films measured under white light illumination in the inert nitrogen atmosphere (0 h) and after exposure to ambient air. The PV device made of P3HT:[70]ThCBM blend exhibits initial power conversion efficiency(*η*) of 0.71% which is reduced, after exposure to ambient air for 6 h, to *η *= 0.37%. The reduction is due to the decrease in short-circuit current (*I*_sc_) from 4.02 to 2.08 mA/cm^2 ^while open-circuit voltage (*V*_oc_) remains 0.55 V. Similar decreases in both power conversion efficiency and *I*_sc _were observed also on the device made of P3HT:[60]ThCBM blend (initial: *η *= 0.86%, *I*_sc _= 4.05 mA/cm^2^; after 6 h: *η *= 0.42%, *I*_sc _= 1.8 mA/cm^2^; *V*_oc _remains 0.48 V). In other words, both blends exhibit similar PV performance as well as its development in time.

It is noticeable that the initial power conversion efficiency of the P3HT:[70]ThCBM blend is lower but its decrease in time is also slightly slower. Based on AFM topography (shown as inset images in Figure 3a, b), both blend films are smooth (RMS roughness around 2 nm) with shallow pits (10-20 nm deep, 100-200 nm in diameter). These pits do not affect the electronic homogeneity of the films. The films appear electronically uniform as observed by KFM [43]. Therefore, to understand the differences in PV performance, we characterized dynamic opto-electronic response by modifying standard KFM technique. We stopped the physical motion of the AFM tip in one direction so that the corresponding axis in the image rep-resents time axis. During scanning in this modified regime we were switching on and off white light illumination repeatedly.

Resulting data (KFM images and their cross sections) are shown in Figure 3c, d. Under the white light illumination, the surface potentials of both blend films shifted. In the case of P3HT:[60]ThCBM blend, the shift of surface potential is slower. This indicates that, after the illumination is switched off, the film keeps negative charge. This behavior has already been observed also on BH PV cells containing PCBM [49] as well as all-polymer cells [50]. It was attributed to electron trapping in shallow traps [51]. Persisting negative charge may then limit the surface potential shift under repeated illumination being switched on. In the case of P3HT:[70]ThCBM, the surface potential comes repeatedly back almost to the starting level during the short term, which indicates that the electron trapping in this blend is minimized, and therefore it is beneficial for PV applications.

The absolute values of the overall surface potentials shifted over time independently on the illumination. This effect is attributed to degradation of the blend films, as the AFM/KFM characterization was performed under ambient conditions. Degradation of the blends was also observed as a decrease in photo-response. There was almost no photo-response observed after 250 min in the case of P3HT:[60]ThCBM blend, and the same state is reached after 1000 min in the case of P3HT:[70]ThCBM.

*I*(*V*) and KFM characteristics indicate that the P3HT:[60]ThCBM degrades much faster than the other one. The difference between the macroscopic and microscopic performance is most likely caused by the presence of the metal electrode (in the macroscopic characterization), which partially seals the organic films. From this point of view, contact-less KFM characterization revealed different opto-electronic properties of the blends, not detected by the macroscopic *I*(*V*) characteristics.

### PPy-diamond heterojunction

**Figure 4:**
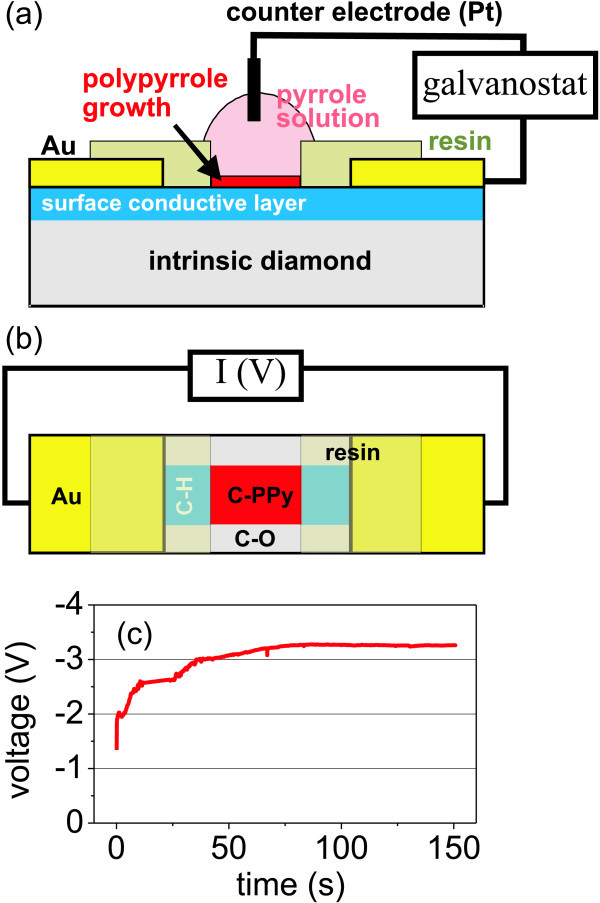
**Design and electrochemical synthesis of PPy-diamond heterojunction**: **(a) **Schematic cross-sectional drawing of experimental setup for electrochemical synthesis of PPy on diamond device structure. **(b) **Schematic top view of PPy-diamond device connected for electrical measurements. **(c) **Voltage as a function of time as detected during the electrochemical synthesis in galvanostatic regime.

For creating organic-inorganic junction, we synthesized and grafted the PPy on diamond electrochemically from pyrrole (0.24 M) and NaCl (0.1 M) aqueous solution by the application of a constant current (current density *-*0.3 mA/cm^2^) and the employment of a hydrogen-terminated intrinsic monocrystalline diamond (synthetic IIIa CVD diamond) with conductive surface as a working electrode [44]. For the electronic transport measurements, we defined H-terminated conductive narrow channel (5 *μ*m wide) on a monocrystalline diamond surface by selective oxygen plasma discharge treatment through a photolithographic mask. PPy was electrochemically synthesized on the channel. Synthesis and electronic measurement setups are shown schematically in Figure 4. Typical PPy growth curve is shown there as well.

**Figure 5:**
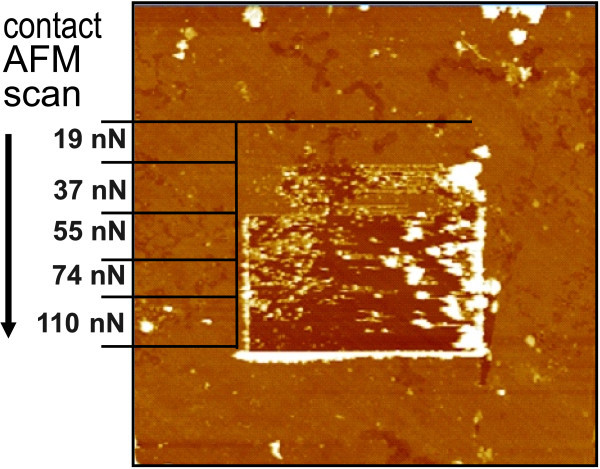
**Scratching of the PPy film by AFM tip with increasing contact force**. Forces reaching 40 nN are strong enough to remove PPy molecules from diamond substrate.

Thickness of the PPy film was 25 ± 5 nm based on the height histogram of tapping-mode AFM image after local nanoshaving of the PPy film in contact mode AFM [44]. Then, we applied contact-mode AFM with increasing contact force. At certain threshold contact force, the sharp tip starts to penetrate and remove the organic film. This transition is evidenced in Figure 5. The threshold force deduced in this manner was about 40 nN. This value is comparable to the values observed on a system consisting of diamond with covalently grafted DNA molecules [20,52]. This indicates that the PPy molecules also establish covalent bonds with H-terminated diamond.

**Figure 6:**
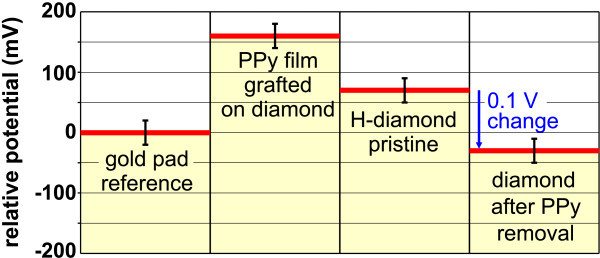
**Average surface potential obtained by KFM on PPy-diamond, H-diamond, and diamond after PPy removal**. The potential values are referenced to the grounded Au contact pad.

Surface potential measured by KFM on the place where the PPy film had been deposited and removed (Figure 6) is significantly lower (by about 0.1 V) than the potential on pristine H-terminated diamond surface. This change is similar to reports on DNA-diamond interfaces [53] and supports the assertion of PPy molecules being linked to diamond surface covalently while removing the H termination. We also observed that the surface conductivity disappeared after the synthesis and the removal of the PPy molecules from diamond surface, which is most likely caused by the missing H termination.

Covalent bonding between PPy and diamond was confirmed also theoretically. Calculated interaction energy of about 6 eV per bond pointed to a covalent character of the bonds formed at the one- and multi-bond contacts between PPy and diamond [54].

Based on the above experimental and theoretical results, we propose a model that, during the electrochemical synthesis of PPy on diamond, the hydrogen atoms are removed and, instead, PPy molecules establish covalent bonds with carbon atoms of diamond.

**Figure 7:**
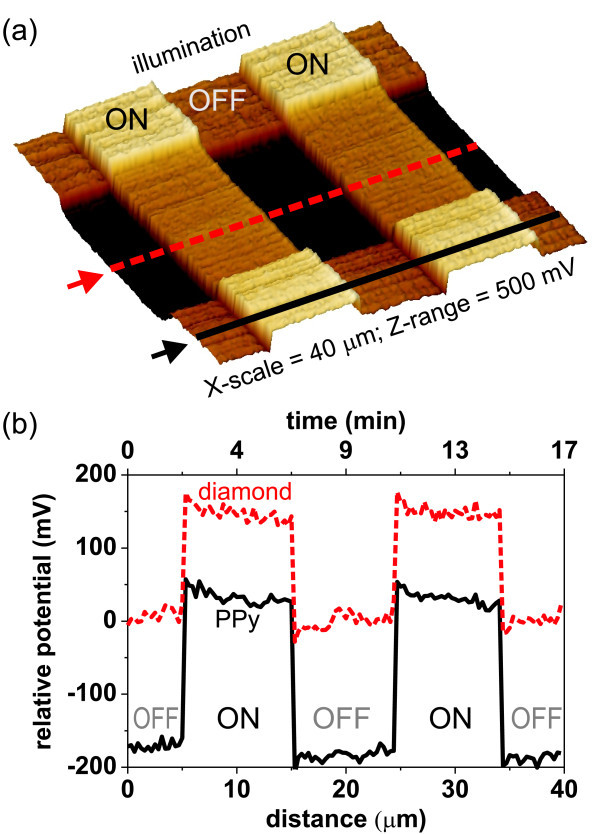
**Surface potential of PPy-diamond heterojunction as function of illumination and time**. **(a) **Three-dimensional representation of surface potential map, and **(b) **temporal potential profiles that were obtained via KFM scanning across PPy-diamond and bare diamond surface (where PPy was removed) under repeated illumination switching. The positions of profiles are indicated by lines and arrows in the image.

To study optoelectronic properties at the PPy-diamond interface we first removed PPy from a small area (10 × 10 *μ*m) using the AFM nanoshaving. Micro-Raman spectroscopy confirmed the removal of PPy [45]. This area and its surroundings were then studied by KFM in the dark and under the white light illumination. Similar to the experiments on advanced bulk heterojunctions, the KFM measurement was performed with one-scan direction disabled, and the illumination was repeatedly switched on and off (Figure 7a). Figure 7b shows temporal potential profiles that were obtained via KFM scanning across PPy-diamond and bare diamond surface (where PPy was removed) under repeated illumination switching. The positions of profiles are indicated by lines and arrows in the image. The changes of potential are relatively fast (*<*1 s, limited by KFM scan speed), reproducible, and well defined.

**Figure 8:**
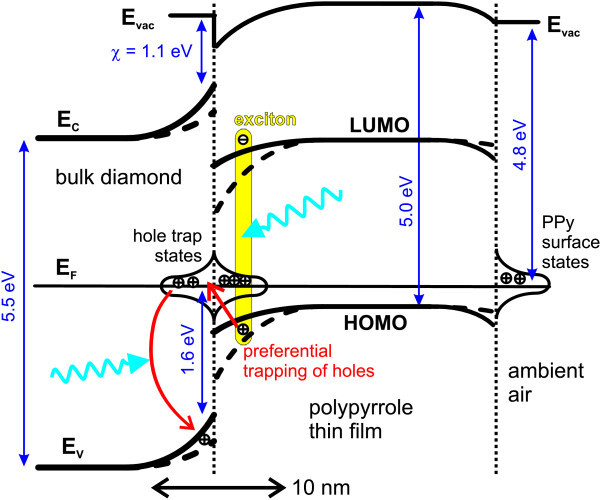
**Energetic scheme of the PPy-diamond system in equilibrium in the dark (full line) and under white light illumination (dashed line)**.

Based on the surface potentials combined with data from the literature for both materials, we were able to assemble the energy band configuration of the PPy-diamond system (Figure 8). Detailed description of the procedure for band diagram assembly is given in [45]. Theoretical calculations showed that, for the PPy-C:H interface, the charge neutrality level is located below the Fermi level, so the transfer of electrons occurs from an electrode to the PPy molecules [54]. This confirm downward band bending of PPy bands at the junction to diamond.

Under illumination, the observed positive shifts of surface potentials correspond to a decrease of work function in both PPy and diamond as indicated in Figure 8. These shifts of surface potentials were attributed to the SPV effects. Based on the SPV theory, a model of charge transfer between diamond and PPy was established [45]. This model suggests that under illumination strongly bound excitons are generated in PPy. The excitons can be split at the interface with diamond, and the holes are trapped in diamond surface/interface states.

**Figure 9:**
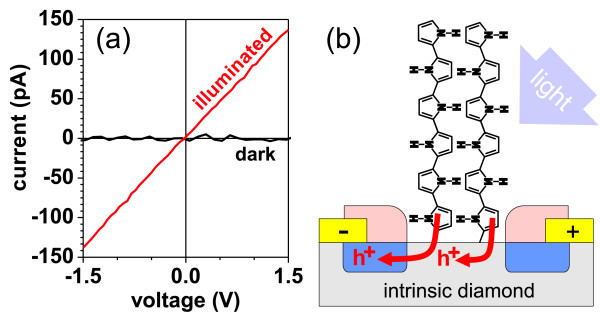
**Electronic transport characteristics and scheme of PPy-diamond hetero-junction**. **(a) **Current-voltage characteristics of the PPy-diamond system in the dark and under white light illumination. **(b) **Conceptual drawing of charge transfer of photogenerated holes from PPy to diamond where they support the in-plane conductivity.

The manner in which these holes contribute also to sub-band gap optical excitation of free holes in diamond depends on many factors [45]. Therefore, we characterized electronic transport properties of the PPy-diamond system by *I*(*V*) measurements both in the dark and under the white light illumination. In the dark, the diamond channel with grafted PPy on top was highly electrically insulating as expected due to the missing H termination. The conductivity does not recover even when we remove PPy and expose the diamond surface to the ambient air (adsorbates) again. Hence, the adsorbates play no role in this effect. Under the white light illumination, the channel with PPy turned electrically conductive (approximately 100 pA when 1 V is applied, see Figure 9a). Such changes of electrical current occur within 1 s and are reproducible [46]. This is in agreement with the fast changes in the surface potential as shown in Figure 7b.

We can explain this illumination-induced conductivity by the charge transfer of holes from PPy to diamond as suggested above. The electronic transport measurements indicate that the holes accumulated in diamond-trap states are excited to diamond valence band, where they support the in-plane conductivity [46]. Such concept is schematically illustrated in Figure 9b. Relatively quick response to the change of illumination can be explained by a quick re-trapping of photogenerated holes in diamond and their recombination with photo-electrons remaining in the vicinity of the PPy-diamond junction due to downward band bending of PPy LUMO. The response rate is comparable with the organic blend [70]ThCBM:P3HT shown in Figure 3c. Also, we suggest that the photo-electrons are not persistently trapped. Hence they can support the quick changes in potential and current. They may also form another transport channel along the heterojunction but this still remains to be investigated.

The observed enhancement of in-plane conductivity under illumination might be explained also by the bridging effect via the semi-conductive and photosensitive PPy. To resolve this issue, we have measured mobility of charge carriers using Hall effect. Mobility of holes in PPy and diamond differs by at least five orders of magnitude (H-terminated diamond: 10^0^-10^2 ^cm^2^/Vs, PPy: 10^-5^-10^-10 ^cm^2^/Vs). We prepared microscopic conductive square (10 × 10 *μ*m^2^) with electric leads at the corners by selective hydrogen and oxygen terminations. A resin layer with the opening at the position of the square has been prepared by UV lithography to restrict the active area for electrochemical deposition. PPy was electrochemically synthesized under similar conditions as described in the previous sections. The Hall voltage was detected by two electrometers, and the current of 2 pA was supplied by Keithley 220 source. The magnetic field applied during the experiment was ± 0.2 T. The mobility was evaluated from the Hall voltages to 7 cm^2^/Vs [47]. However, the Hall voltages were slowly drifting in time, and thus the mobility was obtained with high error bar of 20 cm^2^/Vs.

**Figure 10:**
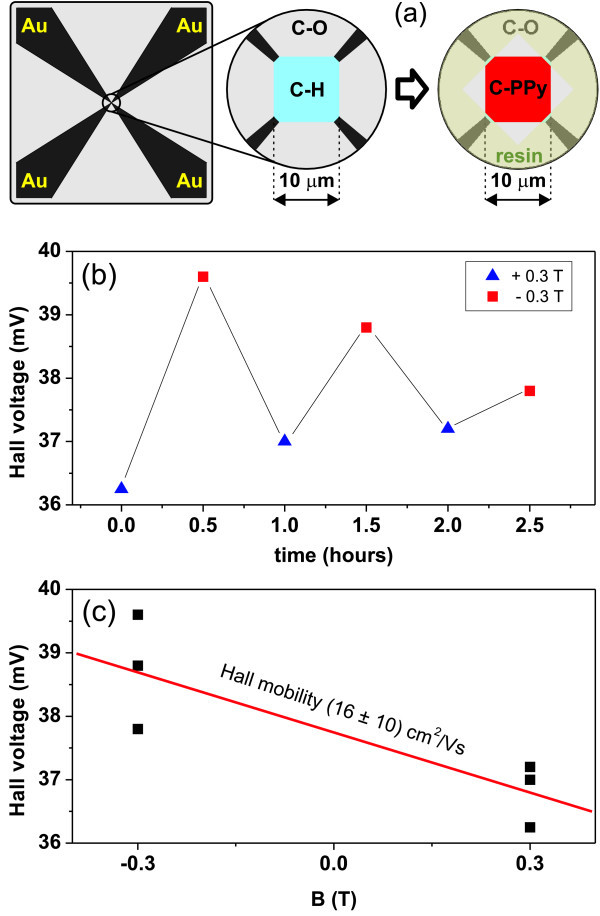
**Hall effect measurements on PPy-diamond heterojunctions**. **(a) **Schematic top view of the diamond in-plane mesa structure for Hall effect measurements showing a bare H-terminated mesa structure and its transformation into PPy-diamond mesa structure. Mesa surroundings are electrically insulated by oxidation of diamond surface. The resin encapsulation is used to confine PPy growth to the mesa area. **(b) **Hall voltage on the PPy-diamond mesa structure measured under +0.3 T (triangles) and - 0.3 T (squares) as a function of time. **(c) **The same Hall voltage plotted as a function of magnetic field. All measurements were done under the cold light illumination.

To reduce the effect of Hall voltage drift, we re-designed electrical connection of the PPy-diamond structure as shown in Figure 10a and also increased the magnetic field. The obtained diagonal Hall voltage under white light illumination (cold light source, 40 klx) is plotted as a function of time in Figure 10b. The Hall voltage is still varying in time, and the difference between values for positive and negative magnetic field, which should correspond to the true Hall voltage, is decreasing. We assign this effect to temperature gradients (up to several kelvins) arising in the measuring chamber because of heat flow from the magnet coils. These gradients create thermal and thermomagnetic electromotoric forces (EMFs) [55] of the order of 1 mV modifying the measured voltage. In more conductive samples, these EMFs can be partially suppressed by alternating the direction of the measuring current. Unfortunately, in our case, the necessary long times of RC relaxation and integration (tens of minutes) prevent a successful application of this procedure. However, after leaving the magnet to cool down to room temperature, the repeated measurement shows a similar dependence, confirming in this way that we really observed the Hall voltage.

The same Hall voltages plotted as a function of magnetic field are shown in Figure 10c. If an arithmetic mean is performed for each applied magnetic field and these values are used for evaluation, then the resulting Hall mobility is 16 ± 10 cm^2^/Vs. This revised value of mobility with much lower relative error supports the idea of charge transport via diamond and thus transfer of charge from PPy to diamond.

## Conclusions

In this article, we showed that employing and combining advanced scanning probe techniques can provide significant insight into the correlation of structural, chemical, and opto-electronic properties of organic-based heterojunctions on the microscopic scale. We presented specific results obtained on fully organic bulk heterostructures and on heterojunction of diamond with electrochemically synthesized organic dye. Although these systems seem to be different, from the optoelectronic point of view, they behave in a similar way. In both systems, strongly bound excitons are created which dissociate at the interface with the other material. Correlation of advanced scanning probe techniques (AFM, KFM, and CS-AFM) with micro-Raman mapping identified the reasons for low PV efficiency and could resolve differences in optoelectronic quality and stability by contact-less monitoring of dynamic photo-response of the organic blends. We demonstrated the applicability of the above methodology also to a hybrid organic-inorganic system. By correlating AFM nanoshaving, KFM surface potentials, as well as *I*(*V*) characteristics, we proved that during the electrochemical synthesis a covalent bond between diamond and PPy is formed. Such conclusion is difficult to make based on commonly applied techniques such as X-ray photoelectron spectroscopy because here we have all-carbon systems (polymer-diamond). Furthermore, based on microscopic KFM and SPV measurements, the model of charge transfer from PPy to diamond under illumination was proposed. The model was supported by in-plane *I*(*V)* and Hall effect measurements.

## Abbreviations

AFM: atomic force microscopy; CS-AFM: current-sensing AFM regime; EMFs: electromotoric forces; ITO: indium tin oxide; KFM: Kelvin force microscopy; PPy: polypyrrole; SPV: surface photovoltage.

## Competing interests

The authors declare that they have no competing interests.

## Authors' contributions

BR conceived the idea of PPy-diamond, designed, and coordinated the study, participated in scanning probe experiments and PPy synthesis, participated in the experimental setup development, participated in discussions and analysis, and prepared the manuscript. JC carried out scanning probe experiments, fabricated device structures, participated in the experimental setup development and discussions, helped in drafting the manuscript. AK provided diamond specimens and their surface terminations. ML carried out micro-Raman spectroscopy and participated in discussions. PH performed Hall measurements and data analysis. JM assisted with Hall measurement and discussions. AP prepared organic blends and measured *I*(*V*) characteristics of the blends. VC participated in the design of the organic blends study and discussion; built up the experimental setup for preparation and macroscopic study of organic blends. AF conceived the scanning probe study of organic blends and the idea of comparing Hall mobility; participated in the experimental setup development. JK participated in discussions of electronic transport and overall results.
